# The Origin of Amerindians and the Peopling of the Americas According to HLA Genes: Admixture with Asian and Pacific People

**DOI:** 10.2174/138920210790886862

**Published:** 2010-04

**Authors:** A. Arnaiz-Villena, C. Parga-Lozano, E. Moreno, C. Areces, D. Rey, P. Gomez-Prieto

**Affiliations:** 1 Department Immunology, University Complutense, The Madrid Regional Blood Center, Madrid, Spain; 2 Department Surgery and Liver Transplant, Hospital 12 de Octubre, Madrid, Spain

**Keywords:** Aleuts, Amerindians, Athabaskans, Eskimo, HLA, peopling of America, mtDNA, Y Chromosome.

## Abstract

The classical three-waves theory of American peopling through Beringia was based on a mixed anthropological and linguistic methodology. The use of mtDNA, Y chromosome and other DNA markers offers different results according to the different markers and methodologies chosen by different authors. At present, the peopling of Americas remains uncertain, regarding: time of population, number of peopling waves and place of peopling entrance among other related issues. In the present review, we have gathered most available HLA data already obtained about First Native American populations, which raise some doubts about the classical three waves of American peopling hypothesis. In summary, our conclusions are: 1) North West Canadian Athabaskans have had gene flow with: a) close neighboring populations, b) Amerindians, c) Pacific Islanders including East Australians and d) Siberians; 2) Beringia was probably not the only entrance of people to America: Pacific Ocean boat trips may have contributed to the HLA genetic American profile (or the opposite could also be true); 3) Amerindians entrance to America may have been different to that of Athabaskans and Eskimos and Amerindians may have been in their lands long before Athabaskans and Eskimos because they present and altogether different set of HLA-DRB1 allele frequencies; 4) Amerindians show very few “particular alleles”, almost all are shared with other Amerindians, Athabaskans and Pacific Islanders, including East Australians and Siberians; 5) Our results do not support the three waves model of American peopling, but another model where the people entrance is not only Beringia, but also Pacific Coast. Reverse migration (America to Asia) is not discarded and different movements of people in either direction in different times are supported by the Athabaskan population admixture with Asian-Pacific population and with Amerindians, 6) HLA variability is more common than allele veriability in Amerindians. Finally, it is shown that gene genealogy analises should be completed with allele frequency analyses in population relatednes and migrations studies.

## INTRODUCTION

The First Amerindian Natives are postulated to have come from Asia through the Bering land bridge between 30,000–12,000 years before the present (BP). These conclusions have been based on cultural, morphological and genetic similarities between American and Asian populations. Both Siberia [1] and Mongolia [2,3] have been put forward as the most likely places of origin in Asia.

Greenberg first postulated the triple migration theory Fig. (**1**) for explaining the peopling of the Americas [4]: Amerindians (most North and South American Indians; 12,000 years BP), Na-Dene (Athabascans, Navajo, Apache; 8,000 years BP) and Eskimo-Aleuts (6,000 years BP). Research carried out before the widespread use of Y Chromosome (Y Chr) and other nuclear DNA markers including mtDNA [5] for the study of populations [6,7] supported the three-wave model. However, other mtDNA studies have not [8,9]; other authors postulate only one wave coming from Mongolia / North China as giving rise to the First Native American ancestors [2,3]. The study of Y Chromosome DNA markers seemed to suggest the existence of a single major paternal haplotype in both North and South American Native populations [10,11]. However, other studies on Y Chromosome show that more than one paternal founder haplotypes arrived in America during different migrations [12], probably from Siberia [13]. See also Fig. (**1**) [14-17].

More recently, new mtDNA analysis has suggested that all mtDNA lineages must have been isolated in Asia before entering the New World by at least 7-15 thousand years. They even suggest that this place must have been Beringia [18]. Also, a dispersal of Amerindians coming from Asia has been put forward through Coastal Pacific line [19] based on all available archaeological, anthropological and mtDNA and genetic data. 

All these calculations are done by using paternal (Y Chr) or maternal (mtDNA) lineages may be biased when populations displacements are concerned, as in the putative Amerindians displacement from Asia to the Americas. In addition, other authors [20] using nuclear histocompatibility (HLA) markers do not regard as important and possible to establish the number and timing of migration waves. The important issue is whether immigrants (Amerindians) were already differentiated (in Asia) into such ethnic groups whose descendants are still to be found in Asia. If they were differentiated then the question of how and when they crossed the Bering Land Bridge is a secondary one [20].

Y Chr and mtDNA studies seem unable to resolve this question, mainly because the studies have been gene- rather than population- based. Their emphasis has been on variant genealogies rather than on population frequencies studies. In this regard HLA data may be more informative [20] because maternal and paternal lineages and both frequencies (i.e.: genetic distances, dendrograms and correspondence analyses) and genealogies (quasi-specific HLA alleles and haplotypes) may be studied for comparing populations.

Alu-insertion investigations have also been carried out to ascertain the origin of First Americans [21]. The results are not concordant with the multiple-wave migration hypothesis; a surprisingly short genetic distance between Chinese and Native Americans was found and explained by a recent gene flow from Asia [21].

A Trans-Pacific route of American peopling from Asia or Polynesia has been suggested because HTLV-1 virus strains shared identical sequences in Japan and in the northern coast of South America [22] and some HLA alleles may have been introduced by the same Trans-Pacific route [15]. Finally, both genetic [17] and archaeological [16] evidence suggests that a two-way Trans-Atlantic traffic occurred before Columbus discovered America Fig. (**1**); archaeologists in New Mexico have recently found tools used 20,000 years ago in Spanish Solutrean culture [16].

All these discrepancies and uncertainties about Amerindian origins may be due to methodological (sampling) differences and also to the different genealogical history of each genetic marker and/or to the phylogenetic usefulness of different DNA markers. For instance, functional molecules — cytochrome (cyt) b mtDNA— are used against an admixture of intronic and exonic DNA markers, as in the Alu or STR studies: the obtained molecular genetics history could not be the same one. In addition, population movements should be studied like a “group of genes” movements, i.e.: with gene frequencies (genetic distances, dendrograms and correspondence analyses), which better reflect a population displacement and other populations (Asian / Amerindian) relatedness, and afterwards completed with genealogies (quasi-specific HLA alleles, HLA haplotypes, mtDNA and Y Chr markers).

Thus, in the present work, we have studied the North, Meso and South American Amerindians’ HLA gene frequencies and compared it with those of other North American Indians and worldwide populations, particularly with Asian and Pacific populations. Also, we have studied the following Amerindian ethnic groups: Seri, Mixe, Mixtecans, Zapotecans, Guaranis [23], Lakota Sioux [24], Mazatecans [25], Teeneks [26], Mayans [27], Kogi, Arsario, Arhuacs, Wayu [28], Cayapa [29], Lamas [30], Aymaras [31], Quechuans [32], Terena Indians [33], Xavantes, Mayos [34], Uros [35], Nahuas [36], Tarahumaras [37], Toba Pilaga, Mataco Wichi, Eastern Toba [15], Mexican Mestizos and Jaidukama (unpublished results) and also Aleuts [14].

Our aims are: 1) To determine the HLA class I (A and B) and class II (DRB1 and DQB1) quasi-specific Amerindian allelic lineages (hereafter ‘‘alleles’’ for simplicity) or specific HLA haplotypes by using DNA sequencing and serology; in other words, the most frequent HLA alleles and haplotypes in Amerindians which do not exist or exist in very low frequency in other populations, i.e.: *genealogy comparisons* and 2) To compare the Amerindians HLA allele frequencies with those of other First American Natives (Na-Dene, Eskimo and Aleuts) and also those of other worldwide populations in order to clarify the still unclear peopling of the Americas and the origins of Amerindians, i.e.: *groups of genes comparisons* by using genetic distances, Neighbor Joining (NJ) dendrograms and correspondence analyses.

## RESULTS AND DISCUSSION

### DRB1 Alleles and HLA-Extended Haplotypes: North-American and Meso / South-American Populations

The low number of class I alleles found may be artificial, since many of them may not have been yet detected. In fact, many more HLA Class I alleles are defined at present day (www.anthonynolan.org.uk).

We have chosen DRB1 alleles because many populations are typed for DRB1 high resolution alleles and very few for HLA class I or other class II loci. No completely specific DRB1 alleles are found in North or South American populations: some of the alleles are found in other populations in a very low frequency Fig. (**2**), footnote, [38]. At the moment, the only exceptions are DRB1*0411 and DRB1*0417 alleles, which are only found in all studied Meso and South Amerindians (Table **1**), Fig. (**2**), bold red color (www.antonynolan.org.uk), compared to previous times.

Notwithstanding, some Meso and South American DRB1 Amerindian alleles tend to be quasi-specific Table **1**, Fig. (**2**), red color, but not North American alleles which are clearly shared with other non-Amerindian populations Fig. (**2**), blue color. This is concordant with the existence of gene flow between Amerindians and Pacific or Siberian people, but not necessarily with a migration of Amerindians from Asia or Pacific Areas, although there are signs of cultural or genetic contacts with Asia [19] or even with Iberians [16,17], Fig. (**1**). DRB1*0802 and DRB1*0407 are present in all the most frequent Meso-American haplotypes Fig. (**2**). DRB1*0802 is also present in Siberian Eskimos and Japanese Ainu: otherwise it is present in almost all Amerindians. DRB1*0407 is present in almost all Amerindian populations and absent or in a non-significant frequency in other populations. DRB1*0403 is present in one South American most frequent haplotype Fig. (**2**) but also is found in high frequency throughout Pacific Islands (Samoa, Papua New Guinea, New Zealand Maories, Taiwan, Tonga, Cook Islands) [38]. A Pacific gene flow in either direction may not be discarded by this genealogy approach also; HLA frequency data (elaborated in dendrograms and correspondence analyses) separate more Amerindians from other populations Figs. (**3**, **4**), see below). DRB1*0407 is an Amerindian allele in one of the most frequent South American Amerindian (Table **1**) [39-57 and previous references], Fig. (**2**), bold red colour [58].

Both genealogy (extended haplotypes, HLA-A,-B,-DRB1,-DQB1) and allele frequency in population analyses (Correspondence and NJ multidimensional populations relatedness) have been carried out.

#### a. North-Americans

The relatively low number of class I alleles found some years ago in Amerindians [57] compared to other worldwide populations may be due to the fact that techniques were not by then detecting new class I alleles and many of the alleles had not yet been described.

Fig. (**2**) shows that the most frequent extended haplotypes in North Americans are specific for North American populations, Yupik (Eskimos), Aleuts and one of the five most frequent haplotypes are also found in Taiwan and Japanese populations (A*24-B*40-DRB1*1401-DQB1*0503). This shows that a low degree of North American haplotypes sharing is found between North American and Asian-Pacific populations. However, there is a clear genetic HLA relatedness between isolated populations close to Beringia: Eskimos, Udegeys, Nivkhs (North East coast of Siberia) and Koryaks and Chukchi from extreme North East Siberia Figs. (**3**, **4**, **5**), and North West American populations: Athabaskan, Alaskan Eskimos (Yupik) and Tlingit.

These results in which class II high resolution alleles and also specific class I-class II extended haplotypes are used suggest that admixture occurred between extreme North East Siberian groups and North American Na-Dene (including Tlingit) and Eskimo (Yupik) people. However, results do not indicate anything about direction of admixture or whether migrations in both directions occurred.

On the other hand, Asian populations which are geographically not close to Beringia (Japanese, Ainu, Manchu, Singapore Chinese, Buyi) do not cluster with North Americans neither in NJ dendrogram Fig. (**3**) or correspondence analysis Fig. (**4**).

Finally, Lakota-Sioux Amerindians which have inhabited in North United States, are not related with Asians and West Siberians Figs. (**3**, **4**, **5**) but with Meso and South Americans.

#### b. Meso-Americans

Most frequent haplotypes Fig. (**2**), relatedness dendrograms Fig. (**3**) and correspondence Fig. (**4**) do not relate these Amerindians with any Asian population, including North East Siberians. Haplotypes of Meso-Americans are shared with other Amerindians and one of them with Alaskan Eskimo (Yupik): A*02-B*35-*DRB1*0802-DQB1 *0402.

#### c. South-Americans

These Amerindian speaking groups are related to other South-American Amerindians and to Meso-American Amerindians Figs. (**3**, **4**). Most frequent haplotypes are shared with other American Amerindians, but not with Asians Fig. (**2**).

In summary, the general view after analyzing the most frequent extended haplotypes (genealogy) is that Amerindians have little relatedness with Asians; this is also confirmed by allele frequencies in populations and the derived analyses, Figs. (**3**, **4**). Genealogy studies are less suitable for comparing and relating groups of people [20]. Also, comparing HLA four loci most frequent extended haplotypes of North-Americans only share one haplotype (A*24-B*40-DRB1*1401-DQB1*0503) with Taiwanese and Japanese in low frequencies see Fig. (**2**) footnote.

### Specific Extended Haplotypes for Amerindian Ethnic Groups and Aleuts

Some new extended four loci haplotypes have been found only in Amerindian and Aleut specific groups and in no other either Amerindians or World populations (Table **2**). It is striking that in small groups of people apparently specific HLA four loci recombinations occur and are fixed. The evolutive forces to achieve an appropriate extended haplotype may be advantageous for a population to deal with its specific environmental pathogens [59]. In this case, evolutive forces to drive *de novo* HLA haplotype appearance must include pathogens and not low frequency genes driven selection. However, both of these types of selection for inducing HLA (or other genes) variability and allele fixation in populations are mathematically indistinguishable [60].

In addition, this high haplotype variability in relatively small ethnic groups is concordant with the fact that HLA haplotype frequencies show greater variation among racial groups than individual alleles [61]. Thus, new allele appearance may be a relatively rare event (usually by a gene conversion mechanism [62]), compared with a new haplotype appearance. In fact, evolution for variability in the MHC may be more frequent in haplotype and not in allele diversification.

See references: [25-27,30-32,34-36,14].

New HLA alleles are continuously being described in populations (Anthony Nolan database: http://www.anthonynolan.org.uk, [38]) but this does not necessarily mean that are continuously being produced; they may have been fixed as low frequency alleles in populations for a long time and only described at present times according to technology advances.

In conclusion, new specific haplotypes are found in North and South American Amerindians, while specific alleles for a particular Amerindian population are rarely found or not found; new alleles are being newly described in a particular population but later found in others. Selection for variability within North and South American Natives is acting upon haplotypes more than upon alleles Fig. (**2**). This finding may be universal for all World populations.

### Genes and Languages

It was postulated that genes correlated with languages [63]; however, from Fig. (**3**) (NJ dendrogram) it may be seen that Na-Dene / Eskimo / Siberian / group is genetically very close as measured by HLA-DRB1 frequencies and speak distant languages [64]; this is confirmed by HLA-DRB1 and HLA-DQB1 correspondence analysis Fig. (**4**). Both NJ and correspondence analysis correlates quite well with geography but does not correlate with languages. Some authors find correlation between genes and languages when selected ethic groups and selected languages are used but only at macro-geographical level.

### mtDNA and Y Chr Markers

Specialists have studied genealogies of haplotypes and / or other markers [5,11,19,18]. However, for studying populations relatedness and migration genetics it is more suitable to study gene frequencies (or allele frequencies) as done in Figs. (**3**, **4**). It is remarkable that Northern USA Lakota-Sioux is genetically close to other Amerindians from South America as expected. However, Canadian Athabaskans go together with Northern Americans First Inhabitants and Siberians Figs. (**3**, **4**).

In general, mtDNA and Y Chr markers have studied the origins of Amerindians [11,19,18], postulated time and place of entrance of Amerindians, Athabaskans and Eskimo [19], and whether the genetic findings fit with Greenberg linguistically based theory [4] (three waves of Americas peopling). Also, archeological findings have been contrasted with genetic findings [1,16,19]. In the end, conclusions are diverse and no consensus exists about Amerindian origin and relatedness [2,3,5,8-13,19].

### HLA Amerindian Genetic Anthropology

HLA has been ignored by anthropologists to study genetics probably because a lack of HLA understanding and the claim that HLA is shaped by selection because of disease linkage; mtDNA and Y Chr (OMIM, http://ncbi.nlm.nih.gov/omim) are also linked to diseases. In addition, they only give a paternal or maternal view of markers genealogy which may have been divergent in small primitive colonizing populations. However, HLA is a nuclear marker giving an even genealogy and genetic history for both sexes. The best test showing that HLA is a good genetic marker for studying population relatedness is that it usually correlates with geography.

HLA dendrograms or correspondence analyses based on HLA frequencies show that Amerindians (in the sense of Greenberg definition, [4]) seem to be separated from other World wide populations, including northern Canadian Athabaskans and Eskimos. The latter cluster in Fig. (**3**) with Siberians. This means that HLA-DRB1 and HLA-DQB1 allele frequencies are completely different in Amerindian compared to from other First American Natives or other World populations.

In addition, the studied populations show particular HLA four loci haplotypes for each specific studied population (Table **2**). This is not the case for HLA-DRB1 alleles: except for two DRB1 alleles —DRB1*0411 and DRB1*0417, Fig. (**2**) Meso and South American Amerindians alleles are shared with the following populations: 1) Siberians, 2) other First American Inhabitants including Athabaskans and Eskimo, but not Aleuts [14], probably because Aleuts come from a Baikal Lake ancient stock that are related to both Aleuts and European Lapps (Saami), 3) Asian Pacific Coast populations (Ainu, Japanese, Taiwan) and to a lesser extent with Indochina people and, 4) East Australian Aborigins and Pacific Islands, like Papua New Guinea or Samoa groups.

In this context and because Canadian Athabaskans have been placed in the postulated entrance for American peopling [4], an haplotype study was done by computing the most frequent Athabaskan two loci haplotypes (DRB1-DQB1) [55] and looking where this part of Chromosome 6 was found around the World (i.e.: a genealogy study, which completes our population frequency study). Results are shown in Table **3** (see footnote). Athabaskans DRB1-DQB1 genes are shared with: 1) Neighbors, including Alaskan Eskimo (Yupik), 2) Amerindians from North and South America, 3) Siberians, 4) Pacific Islands inhabitants, including those of Samoa, Papua New Guinea, Cook Islands and Japanese-Ainu and even Eastern Australia Aborigins. Only one haplotype (f), in Table **3** is exclusively shared among Amerindians. This suggests that Athabaskans are composed of a genetic HLA admixture and that gene flow has occured between Athabaskans and all the other above mentioned Pacific-Asian populations. The direction of the hypothetical HLA gene flow is not known and may have occured in different directions in different times. Thus, there is no point to conclude about one or more waves of Americas peopling from our data. However, this also shows that not only Beringia was an active pass of primitive Amerindians, but also Pacific navigation was.

Results on North American and South American population HLA alleles also support this view Fig. (**2**). Why nowadays North and South American Amerindians are altogether different populations to the rest of the World regarding to HLA frequencies is only a matter of speculation:

- It has been calculated that about 80 million First American Natives died after 1492 AD within the following 100 years from Alaska to Patagonia [65]. It was mainly due to a lack of appropriate immune response to European-borne diseases, mainly measles, influenza and plague [65]. This may have shaped the First American Natives HLA profile by increasing rare HLA alleles able to present new pathogens to T cells [60,61,66]. However, this is not likely since First North American Natives (non-Amerindians) also suffered many epidemics [65] and do not have as different HLA profile from Asians, as Amerindians do.
- First American Natives, including Alaskan Eskimo (Yupik) and Athabaskans must have been in America long before calculated (20,000 years ago), because both North and South American Fist Natives were similarly susceptible to European-borne diseases [65], indicating the existence of long population isolation.

## CONCLUSIONS

1. North West Canadian Athabaskans have had gene flow with a) close neighboring populations, b) Amerindians, c) Pacific Islanders including East Australians and d) Siberians.
2. Beringia was not probably the only entrance of people to Americas: Pacific Ocean boat trips may have contributed to the HLA genetic American profile (or the opposite could also be true).
3. Amerindians entrance to America may have been different to that of Athabaskans and Eskimos and Amerindians may have been in their lands long before Athabaskans and Eskimos because they present and altogether different sets of HLA-DRB1 frequencies.
4. Amerindians show very few “particular alleles”, almost all are shared with other Amerindians, Athabaskans, Pacific Islanders, including East Australians and Siberians. However, specific Amerindian haplotypes are found in isolates.
5. Genes and languages do not correlate.
6. Our results do not support the three-wave model of American peopling, but another model where the people entrance is not only Beringia, but also Pacific Coast. Reverse migration (America to Asia) is not discarded and different movements of people in either direction in different times are supported by the Athabaskan population admixture with Asian-Pacific population and with Amerindians.

## Figures and Tables

**Fig. (1):**
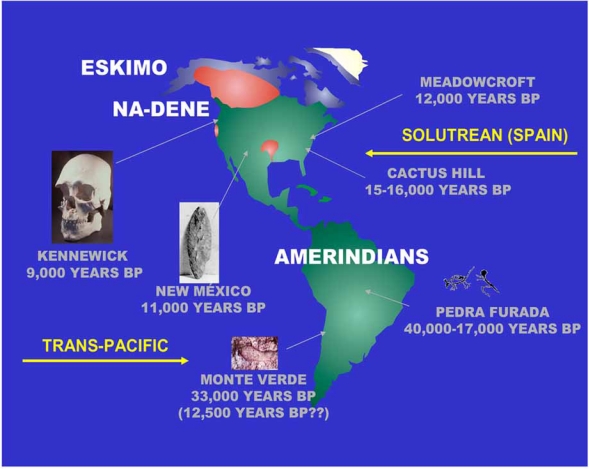
A map of the Americas showing the most accepted theory of peopling of this continent from Asia though Bering Strait [4]. Amerindians (30,000-12,000 years before present, BP); Na-Dene (8,000 years BP), Athabaskans in Canada, Californian Indian isolates and Navajo and Apache from Southern United States; Eskimo (6,000 years BP). Aleuts from Aleutian Islands in Bering Strait are considered separate from Eskimo in linguistic and other anthropological parameters and were present in the Islands before Eskimos reached North America; in addition, Aleut HLA profile is different from Eskimo profile (see text) [14]. Other theories of peopling of Americas are (arrows): 1) Trans-Pacific (from Australia-Pacific Islands [15], and from Iberian Peninsula Solutrean people [16,17]. Archeological relevant findings are also represented (Introduction section references, particularly [16]). Kennewick man from Washington State, USA; Meadowcroft (Pensylvania, USA); Cactus Hill (Virginia, USA); Pedra Furada (Brasil); Monte Verde (Chile).

**Fig. (2).**
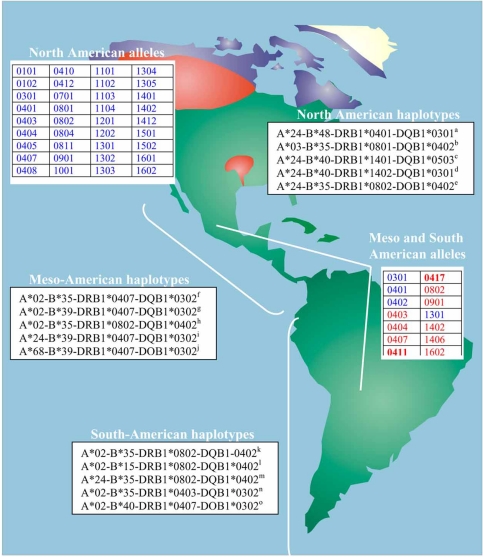
Geography of the most frequent HLA-DRB1 alleles and HLA extended haplotypes (the latter are ordered by frequency) in indigenous populations of America. Most frequent North American DRB1 alleles are represented in blue. “Specific alleles” are highlighted in red. Specific alleles in fact, are “quasi-specific” alleles (see [58]) being found in a very low frequency in Amerindian neighbouring populations, other North American Amerindians, Pacific Islanders, Eskimos, Athabaskans, East Asians (Ainu, Japanese). DRB1*0411 and DRB1*0417 are found in Meso and South American Amerindians (represented in bold red colour). ^a^Yupik (9.3%); Aleuts (6.9%). ^b^Aleuts (8.3%). ^c^Aleuts (6.9%); Yupik (5.4%); Taiwan (6.0%); Inuit and Japanese. ^d^Yupik (6.6%). ^e^Yupik (6.0%). ^f^Seri (18.2%); Teeneks (15.5%); Mayans (10.6%); Mayos (7.3%); Mixtecans (3%); Mazatecans (2.5%); Aymaras (1.7%); Peruvians (1.7%). ^g^Mazatecans (10.8%); Mixe (9%); Mayans (4.2%); Teeneks (3.7%); Terena Indians (2.3%). ^h^Aymaras (10.4%); Mayans (8.4%); Nahuas (6.1%); Mixtecans (6%); Tarahumara (3.4%); Seri (4.5%); Yupik (3.1%); Zapotecans (3%); Mixe (1.5%). ^i^Mayos (8.2%); Mazatecans (3.3%). ^j^Mayans (6.4%); Teeneks (5.2%). ^k^Uros (13.5%); Aymaras (10.4%); Peruvians (9.6%); Mayans (8.4%); Quechuas (6.5%); Nahuas (6.1%); Mixtecans (6%); Seri (4.5%); Tarahumara (3.4%); Zapotecans (3%); Mixe (1.5%). ^l^Ainu (8.0%); Quechuas (4.3%); Mayans (0.7%). ^m^Uros (6.8%); Mixtecans (5%); Mayans (4.2%); Teeneks (3.7%); Aymaras (3.1%); Lamas (2.4%); Seri (2.3%); Terena Indians (2.3%); Quechuans (2.2%). ^n^Uros (6.3%); Quechuans (2.9%); Mayans (0.7%). ^o^Lamas (5.9%); Aymaras (2.3%) Mayans (0.7%). See references: [14,25-27,30-37,58]. It was considered for figure elaboration: 1) Most frequent extended four loci haplotypes in North America were taken from Aleuts [14]; Lakota-Sioux [24] and Yupik [58]. The five most frequent ones were chosen from each population; 2) Most frequent DRB1 alleles in North America were taken (all) from Athabaskans, Canadian Penutians, Tlingit [55], Lakota-Sioux [24], Yupik [58], Aleuts [14] and Zuni. They are represented in blue color; 3) Most frequent extended four loci haplotypes in Meso and South America were taken from Arnaiz-Villena *et al.* papers (Table **1**); 4) Most frequent DRB1 alleles in Meso and South American Amerindians were taken from Table **1** (Arnaiz-Villena *et al.* papers) and see [58]. Highest frequencies DRB1 alleles found were: DRB1*0301, 0401, 0402, 0403, 0404, 0407, 0411, 0417, 1301, 1402, 1406, 1602, 0802 and 0901.

**Fig. (3):**
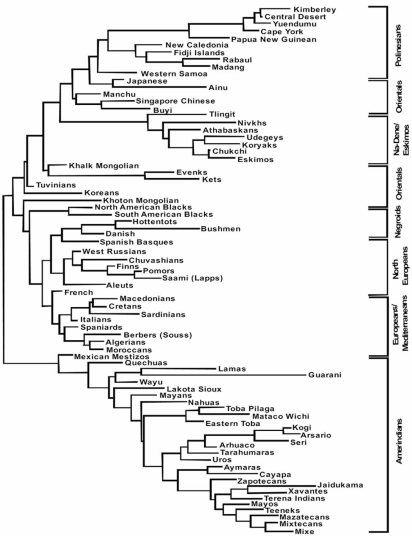
Neighbor-Joining dendrogram based on HLA-DRB1 allele frequencies. The genetic relatedness among Amerindians, Na-Dene, Eskimos, Asians, Negroids, Europeans and Polynesians are determined by calculating the genetic distances between populations (DA), using HLA-DRB1 allele frequencies. Amerindians cluster together and separated from the rest of the World populations [14,34,35,37].

**Fig. (4):**
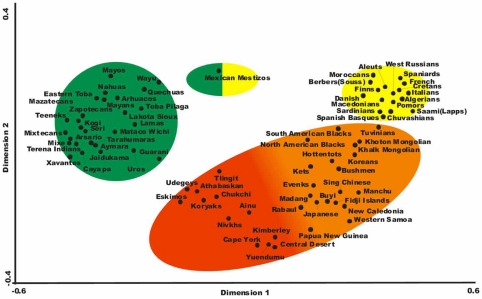
Correspondence analysis based on HLA-DRB1 and HLA-DQB1 allele frequencies. The analysis shows a global view of the genetic relationships among Amerindian, Na-Dene, Eskimo, Asian, Negroid and European populations according to HLA-DRB1 and HLA-DQB1 allele frequencies. These relationships are calculated in *n* dimensions and represented in two dimensions. Circles represent an approximate grouping of populations [14,34,35,37].

**Fig. (5):**
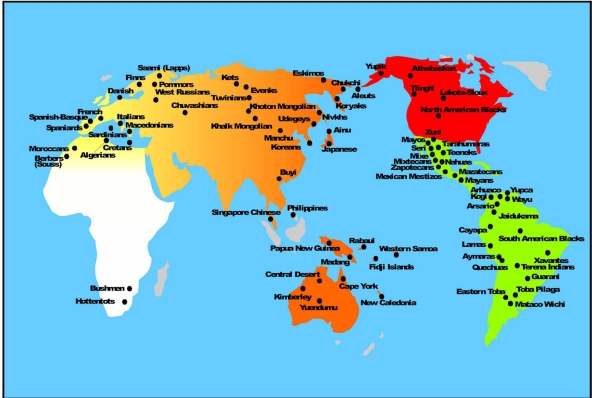
**Map of relevant Amerindian and Asian populations.** A smooth relatedness gradient is reflected by grey intensity. It remarks the results obtained in (Fig. **3**) and (Fig. **4**): Amerindians (homogeneous grey) are separated from the rest of the world by using HLA-DRB1 and DQB1 allele frequencies (North American Zuni and Lakota-Sioux Amerindians cluster together with Meso and South American Amerindians). However, North American Eskimo (Yupik), Athabaskan and Tlingit cluster with Siberian close-by populations (Eskimo, Chukchi, Koryaks, Nivkhs, Udegys). Aleuts are also related to Baikal Area populations and to Laps (Saami, [14]). See Table **1** for population references. See also [14,34,35,37].

**Table 1:** Populations Studied in the Present Work. A Total of 14,698 Chromosomes were Analyzed

Population	N	Reference	Population	N	Reference
Seri	100	[23]	Manchu	50	[39]
Mixe	55	[23]	Koreans	100	[39]
Mixtecans	103	[23]	Japanese	493	[39]
Zapotecans	75	[23]	Ainu	50	[40]
Lakota Sioux	302	[24]	Khalk Mongolians	100	[41]
Mazatecans	90	[25]	Tuvinians	190	[42]
Teeneks	44	[26]	Khoton Mongolians	85	[41]
Mexican Mestizos	99	Unpublished results	Sardinians	91	[39]
Mayans	132	[27]	Italians	284	[39]
Wayu	88	[28]	French	179	[39]
Arhuaco	107	[28]	Spaniards	176	[43]
Kogi	42	[28]	Spanish Basques	82	[43]
Arsario	18	[28]	Algerians	106	[44]
Jaidukama	39	Unpublished results	Berbers (Souss)	98	[45]
Cayapa	100	[29]	Moroccans	96	[46]
Lamas	83	[30]	Macedonians	172	[47]
Aymaras	102	[31]	Cretans	135	[48]
Quechuas	80	[32]	Finns	157	[14]
Xavantes	74	[15]	Saami (Lapps)	81	[14]
Terena Indians	60	[33]	Aleuts	85	[14]
Guarani	32	[23]	Pomors	73	[14]
Toba-Pilaga	19	[15]	Danish	124	[39]
Mataco-Wichi	49	[15]	West Russians	200	[49]
Eastern Toba	135	[15]	Chuvashians	82	[50]
Mayos	60	[34]	Fidji Islands	57	[51]
Tarahumaras	44	[37]	Papua New Guinean	65	[51]
Uros	105	[35]	Central Desert	152	[52]
Nahuas	85	[36]	Yuendumu	119	[52]
Eskimos	80	[53]	Kimberley	82	[54]
Athabaskans	124	[55]	Western Samoa	51	[56]
Tlingit	53	[39]	Madang	65	[51]
Nivkhs	32	[53]	Rabaul	60	[51]
Udegeys	25	[53]	New Caledonia	65	[51]
Koryaks	92	[53]	Cape York	80	[54]
Chukchi	59	[53]	South American Blacks	59	[39]
Kets	22	[53]	North American Blacks	447	[39]
Evenks	35	[53]	Hottentots	65	[39]
Singapore Chinese	71	[39]	Bushmen	103	[39]
Buyi	70	[39]			

**Table 2:** Extended Haplotypes Only Found in Some Amerindian Populations [14,25-27,30-32,34-36]

HAPLOTYPE	(Freq. %)	POPULATION
A*02-B*39-DRB1*1602-DQB1*0301	(3.3%)	Mazatecans
A*02-B*62-DRB1*1602-DQB1*0301	(3.3%)	Mazatecans
A*02-B*15-DRB1*0404-DQB1*0302	(1.5%)	Mayans
A*02-B*39-DRB1*0802-DQB1*0402	(3.4%)	Aymaras
A*02-B*39-DRB1*0901-DQB1*0303	(3.4%)	Aymaras
A*02-B*48-DRB1*0403-DQB1*0302	(7.8%)	Lamas
A*02-B*48-DRB1*0804-DQB1*0402	(7.8%)	Lamas
A*02-B*39-DRB1*1402-DQB1*0301	(3.6%)	Lamas
A*66-B*41-DRB1*1303-DQB1*0301	(3.6%)	Lamas
A*02-B*48-DRB1*0411-DQB1*0302	(2.4%)	Lamas
A*24-B*15-DRB1*0901-DQB1*0303	(1.8%)	Lamas
A*33-B*38-DRB1*1104-DQB1*0301	(1.8%)	Lamas
A*68-B*35-DRB1*0802-DQB1*0402	(3.6%)	Quechua
A*02-B*48-DRB1*1402-DQB1*0301	(2.9%)	Quechua
A*02-B*48-DRB1*0802-DQB1*0402	(2.2%)	Quechua
A*02 B*52 DRB1*0411 DQB1*0302	(3.7%)	Teeneks
A*68 B*35 DRB1*1402 DQB1*0301	(2.8%)	Teeneks
A*68 B*40 DRB1*1602 DQB1*0301	(2.6%)	Teeneks
A*68 B*35 DRB1*1406 DQB1*0301	(2.6%)	Teeneks
A*02-B*35-DRB1*1406-DQB1*0301	(4.2%)	Mayos
A*02-B*48-DRB1*0404-DQB1*0302	(3.3%)	Mayos
A*24-B*51-DRB1*0407-DQB1*0302	(3.3%)	Mayos
A*02-B*08-DRB1*0407-DQB1*0302	(2.5%)	Mayos
A*30-B*49-DRB1*1001-DQB1*0501	(7.5%)	Nahuas
A*02-B*52-DRB1*1402-DQB1*0301	(2.7%)	Nahuas
A*68-B*61-DRB1*1602-DQB1*0303	(2.0%)	Nahuas
A*24-B*15-DRB1*1402-DQB1*0301	(3.2%)	Uros
A*68-B*35-DRB1*0403-DQB1*0302	(3.2%)	Uros
A*24-B*48-DRB1*0403-DQB1*0302	(2.2%)	Uros
A*02-B*40-DRB1*0101-DQB1*0501	(5.6%)	Aleuts
A*24-B*37-DRB1*0801-DQB1*0402	(4.2%)	Aleuts
A*24-B*39-DRB1*0404-DQB1*0302	(4.2%)	Aleuts
A*24-B*39-DRB1*1201-DQB1*0301	(4.2%)	Aleuts
A*02-B*15-DRB1*0401-DQB1*0301	(2.8%)	Aleuts
A*02-B*51-DRB1*1501-DQB1*0602	(2.8%)	Aleuts
A*26-B*40-DRB1*1401-DQB1*0503	(2.8%)	Aleuts
A*32-B*44-DRB1*0701-DQB1*02	(2.8%)	Aleuts
A*68-B*40-DRB1*0404-DQB1*0302	(2.8%)	Aleuts
A*68-B*40-DRB1*0802-DQB1*0402	(2.8%)	Aleuts
A*68-B*39-DRB1*1201-DQB1*0301	(2.8%)	Aleuts

**Table 3:** DRB1-DQB1 Haplotypes also Found in Athabaskans [55] and also in other Populations

DRB1	DQB1	FREQ. (%)
1402	0301	34.7^a^
1401	0503	16.9^b^
0901	0303	10.5^c^
0403	0302	9.6^d^
1201	0301	8.9^e^
0802	0402	4.8^f^
0410	0402	4.0^g^

a aXavantes (25.7%); Mataco Wichi (22.4%); Alaska Yupik Natives (22.0%); Tarahumara (11.9%); Russia Siberia Eskimos Chukotka Paninsula (11.3%).

b Ainu (20.0%); Spain Malaga Gipsy (9.5%); Slovenia (7.9%); Papua New Guinea New Britain Tolai (7.6%); Aleuts (6.9%); Alaska Yupik Natives (6.7%).

c Japan (29.5%); Russia Siberian Khabarovsk Evenki (26.0%); Samoa (25.9%); Russia Siberia Nganasan Dudinka (25.0%); Russia Siberia Koryaks North East Kamchatka (22.3%); Russia Siberia Negidal (20.0%); Russia Siberia Udegeys (19.0%); Russia Siberia Ulchi (15.8%); Russia Siberia Chukchi (14.7%).

d Samoa (17.2%); Russia Siberia Nganasan (12.5%); Taiwan Aboriginal (9.5%); Yucpa (9.3%); Russian Siberia Kushun (8.0%); Lamas (7.8%); Russia Siberia Khabarovsk Evenki (6.0%); Russia Siberia Negidal (5.7%); Philippines (5.2%); Zapotecans (5.2%).

e Russia Siberia Ket Lower Yenisey (17.7%); Russia Siberia Irkutsk Tofalar (17.4%); Russia Tuva (13.7%); Russia Siberia Kets Sulamai (13.6%); Russia Siberia Ulchi (13.0%); Russia Siberia Nganasan Dudinka (12.5%); Russia Siberia Kets Sulamai (11.4%); Russia Siberia Khanty Mansi (8.8%).

f Mixe (28.0%); Tarahumara (27.9%); Xavantes (23.0%); Mixtecans (21.6%); Zapotecans (21.5%); Eastern Toba (18.9%); Jalisco Mestizos (14.9%); Highlands Mestizos (13.8%); Alaska Yupik Natives (13.3%); Toba Pilaga (10.5%); Aymaras (10.4%); Ainu (10.0%).

g Japan (3.8%); Alaska Yupik Natives (3.6%); Alaska Yupik Natives (3.5%); Australia New South Wales Aborigines (2.4%); Papua New Guinea (2.3%); Ainu (2.0%), China (1.8%); Japan (1.7%); Japan Central (1.6%); Mixtecans (1.5%).

These footnote frequencies were taken from reference [38] and from our own publications (Table 1).

## ABBREVIATIONS

BP: = Before present
Cyt: = Cytochrome
HLA: = Human leucocitary antigen
HTLV-1: = Human T-lymphotrophic virus
mtDNA: = Mitochondrial deoxyribonucleic acid
NJ: = Neighbor joining
STR: = Short tandem repeat
Y Chr: = Y chromosome

## References

[1] Crawford MH (1998). The Origins of Native Americans: evidence from anthropological genetics. Cambridge.

[2] Kolman CJ, Sambuughin N, Bermingham E (1996). Mitochondrial DNA analysis of Mongolian populations and implications for the origin of New World founders. Genetics.

[3] Merriwether DA, Hall W W, Vahlne A, Ferrell R E (1996). mtDNA variation indicates Mongolia may have been the source for the founding population for the New World. Am. J. Hum. Genet.

[4] Greenberg JH, Turner C G, Zegura S L (1986). The settlement of the Americas: a comparison of the linguistic, dental and genetic evidence. Curr. Anthropol.

[5] Wallace DC, Torroni A (1992). American Indian prehistory as written in the mitochondrial DNA: a review. Hum. Biol.

[6] Cavalli-Sforza LL, Menozzi P, Piazza A (1994). The history and geography of human genes. Princeton.

[7] Parham P, Ohta T (1996). Population biology of antigen presentation by MHC class I molecules. Science.

[8] Horai S, Kondo R, Nakagawa-Hattori Y, Hayashi S, Sonoda S, Tajima K (1993). Peopling of the Americas, founded by four major lineages of mitochondrial DNA. Mol. Biol. Evol.

[9] Torroni A, Sukernik R I, Schurr T G, Starikorskaya Y B, Cabell M F, Crawford M H, Comuzzie A G, Wallace D C (1993). mtDNA variation of aboriginal Siberians reveals distinct genetic affinities with Native Americans. Am. J. Hum. Genet.

[10] Santos FR, Rodriguez-Delfin L, Pena S D, Moore J, Weiss K M (1996). North and South Amerindians may have the same major founder Y chromosome haplotype. Am. J. Hum. Genet.

[11] Karafet T, Zegura S L, Vuturo-Brady J, Posukh O, Osipova L, Wiebe V, Romero F, Long J C, Harihara S, Jin F, Dashnyam B, Gerelsaikhan T, Omoto K, Hammer M F (1997). Y chromosome markers and Trans-Bering Strait dispersals. Am. J. Phys. Anthropol.

[12] Karafet TM, Zegura S L, Posukh O, Osipova L, Bergen A, Long J, Goldman D, Klitz W, Harihara S, de Knijff P, Wiebe V, Griffiths R C, Templeton A R, Hammer M F (1999). Ancestral Asian source(s) of new world Y-chromosome founder haplotypes. Am. J. Hum. Genet.

[13] Santos FR, Pandya A, Tyler-Smith C, Pena S D, Schanfield M, Leonard W R, Osipova L, Crawford M H, Mitchell R J (1999). The central Siberian origin for native American Y chromosomes. Am. J. Hum. Genet.

[14] Moscoso J, Crawford M H, Vicario J L, Zlojutro M, Serrano-Vela JI, Reguera R, Arnaiz-Villena A (2008). HLA genes of Aleutian Islanders living between Alaska (USA) and Kamchatka (Russia) suggest a possible southern Siberia origin. Mol. Immunol.

[15] Cerna M, Falco M, Friedman H, Raimondi E, Maccagno A, Fernandez-Vina M, Stastny P (1993). Differences in HLA class II alleles of isolated South American Indian populations from Brazil and Argentina. Hum. Immunol.

[16] Holden C (1999). Were Spaniards among the first Americans?. Science.

[17] Bruges-Armas J, Martinez-Laso J, Martins B, Allende L, Gomez-Casado E, Longas J, Varela P, Castro M J, Arnaiz-Villena A (1999). HLA in the Azores Archipelago: possible presence of Mongoloid genes. Tissue Antigens.

[18] Mulligan CJ, Kitchen A, Miyamoto MM (2008). Updated three-stage model for the peopling of the Americas. PLoS One.

[19] Goebel T, Waters MR, O'Rourke DH (2008). The late Pleistocene dispersal of modern humans in the Americas. Science.

[20] Uinuk-Ool TS, Takezaki N, Sukernik RI, Nagl S, Klein J (2002). Origin and affinities of indigenous Siberian populations as revealed by HLA class II gene frequencies. Hum. Genet.

[21] Novick GE, Novick C C, Yunis J, Yunis E, Antunez de May-olo P, Scheer W D, Deininger P L, Stoneking M, York D S, Batzer M A, Herrera R J (1998). Polymorphic Alu insertions and the Asian origin of Native American populations. Hum. Biol.

[22] Leon-S FE, Ariza-Deleon A, Leon-S M E, Ariza C (1996). Peopling the Americas. Science.

[23] Petzl-Erler ML, Gorodezky C, Layrisse Z, Charron D (1997). Funtional and medical implications. Genetic diversity of HLA.

[24] Leffell MS, Fallin M D, Hildebrand W H, Cavett J W, Igle-hart B A, Zachary A A (2004). HLA alleles and haplotypes among the Lakota Sioux: report of the ASHI minority workshops, part III. Hum. Immunol.

[25] Arnaiz-Villena A, Vargas-Alarcon G, Granados J, Gomez-Casado E, Longas J, Gonzales-Hevilla M, Zuniga J, Salgado N, Hernandez-Pacheco G, Guillen J, Martinez-Laso J (2000). HLA genes in Mexican Mazatecans, the peopling of the Americas and the uniqueness of Amerindians. Tissue Antigens.

[26] Vargas-Alarcon G, Hernandez-Pacheco G, Moscoso J, Perez-Hernandez N, Murguia LE, Moreno A, Serrano-Vela J I, Granados J, Arnaiz-Villena A (2006). HLA genes in Mexican Teeneks: HLA genetic relationship with other worldwide populations. Mol. Immunol.

[27] Gomez-Casado E, Martinez-Laso J, Moscoso J, Zamora J, Martin-Villa M, Perez-Blas M, Lopez-Santalla M, Lucas-Gramajo P, Silvera C, Lowy E, Arnaiz-Villena A (2003). Origin of Mayans according to HLA genes and the uniqueness of Amerindians. Tissue Antigens.

[28] Yunis JJ, Ossa H, Salazar M, Delgado M B, Deulofeut R, de la Hoz A, Bing D H, Ramos O, Yunis E J, Yunis E J (1994). Major histocompatibility complex class II alleles and haplotypes and blood groups of four Amerindian tribes of northern Colombia. Hum. Immunol.

[29] Titus-Trachtenberg EA, Rickards O, De Stefano G F, Erlich H A (1994). Analysis of HLA class II haplotypes in the Cayapa Indians of Ecuador: a novel DRB1 allele reveals evidence for convergent evolution and balancing selection at position 86. Am. J. Hum. Genet.

[30] Moscoso J, Seclen S, Serrano-Vela J I, Villena A, Martinez-Laso J, Zamora J, Moreno A, Ira-Cachafeiro J, Arnaiz-Villena A (2006). HLA genes in Lamas Peruvian-Amazonian Amerindians. Mol. Immunol.

[31] Arnaiz-Villena A, Siles N, Moscoso J, Zamora J, Serrano-Vela J I, Gomez-Casado E, Castro M J, Martinez-Laso J (2005). Origin of Aymaras from Bolivia and their relationship with other Amerindians according to HLA genes. Tissue Antigens.

[32] Martinez-Laso J, Siles N, Moscoso J, Zamora J, Serrano-Vela J I, Ira-Cachafeiro J, Castro M J, Serrano-Rios M, Arnaiz-Villena A (2006). Origin of Bolivian Quechua Amerindians: their relationship with other American Indians and Asians according to HLA genes. Eur. J. Med. Genet.

[33] Lazaro AM, Moraes M E, Marcos C Y, Moraes J R, Fer-nandez-Vina M A, Stastny P (1999). Evolution of HLA-class I compared to HLA-class II polymorphism in Terena, a South-American Indian tribe. Hum. Immunol.

[34] Arnaiz-Villena A, Moscoso J, Granados J, Serrano-Vela J I, de la Peña A, Reguera R, Ferri A, Seclen E, Izaguirre R, Perez-Hernandez N, Vargas-Alarcon G (2007). HLA genes in Mayos population from Northeast Mexico. Curr. Genomics.

[35] Arnaiz-Villena A, Gonzalez-Alcos V, Moscoso J, Reguera R, Barbolla L, Ferri A, Fernandez-Perez C, Abd-El-Fatah S, Serrano-Vela JI (2009). HLA genes in Uros from Peru: Origin and relationships with other Amerindians and worldwide populations. Int. J. Immunogenet.

[36] Vargas-Alarcon G, Moscoso J, Martinez-Laso J, Rodriguez-Perez J M, Flores-Dominguez C, Serrano-Vela J I, Moreno A, Granados J, Arnaiz-Villena A (2007). Origin of Mexican Nahuas (Aztecs) according to HLA genes and their relationships with worldwide populations. Mol. Immunol.

[37] Garcia-Ortiz JE, Sandoval-Ramirez L, Rangel-Villalobos H, Maldonado-Torres H, Cox S, Garcia-Sepulveda C A, Figuera L E, Marsh S G, Little A M, Madrigal J A, Moscoso J, Arnaiz-Villena A, Arguello J R (2006). High-resolution molecular characterization of the HLA class I and class II in the Tarahumara Amerindian population. Tissue Antigens.

[38] Middleton D, Menchaca L, Rood H, Komerofsky R (2009). New allele frequency database. http://www.allelefrequencies.net. Tissue Antigens.

[39] Imanishi T, Akaza T, Kimura A, Tokunaga K, Gojobori T, Tsuji K, Aizawa M, Sasazuki T (1992). HLA 1991.

[40] Bannai M, Tokunaga K, Imanishi T, Harihara S, Fujisawa K, Juji T, Omoto K (1996). HLA class II alleles in Ainu living in Hidaka District, Hokkaido, northern Japan. Am. J. Phys. Anthropol.

[41] Munkhbat B, Sato T, Hagihara M, Sato K, Kimura A, Munkhtuvshin N, Tsuji K (1997). Molecular analysis of HLA polymorphism in Khoton-Mongolians. Tissue Antigens.

[42] Martinez-Laso J, Sartakova M, Allende L, Konenkov V, Moscoso J, Silvera-Redondo C, Pacho A, Trapaga J, Gomez-Casado E, Arnaiz-Villena A (2001). HLA molecular markers in Tuvinians: a population with both Oriental and Caucasoid characteristics. Ann. Hum. Genet.

[43] Martinez-Laso J, de Juan D, Martinez-Quiles N, Gomez-Casado E, Cuadrado E, Arnaiz-Villena A (1995). The contribution of the HLA-A, -B, -C and -DR, -DQ DNA typing to the study of the origins of Spaniards and Basques. Tissue Antigens.

[44] Arnaiz-Villena A, Benmamar D, Alvarez M, Diaz-Campos N, Varela P, Gomez-Casado E, Martinez-Laso J (1995). HLA allele and haplotype frequencies in Algerians. Relatedness to Spaniards and Basques. Hum. Immunol.

[45] Izaabel H, Garchon H J, Caillat-Zucman S, Beaurain G, Akhayat O, Bach J F, Sanchez-Mazas A (1998). HLA class II DNA polymorphism in a Moroccan population from the Souss, Agadir area. Tissue Antigens.

[46] Gomez-Casado E, del Moral P, Martinez-Laso J, Garcia-Gomez A, Allende L, Silvera-Redondo C, Longas J, Gonzalez-Hevilla M, Kandil M, Zamora J, Arnaiz-Villena A (2000). HLA genes in Arabic-speaking Moroccans: close relatedness to Berbers and Iberians. Tissue Antigens.

[47] Arnaiz-Villena A, Dimitroski K, Pacho A, Moscoso J, Gomez-Casado E, Silvera-Redondo C, Varela P, Blagoevska M, Zdravkovska V, Martinez-Laso J (2001). HLA genes in Macedonians and the sub-Saharan origin of the Greeks. Tissue Antigens.

[48] Arnaiz-Villena A, Iliakis P, Gonzalez-Hevilla M, Longas J, Gomez-Casado E, Sfyridaki K, Trapaga J, Silvera-Redondo C, Matsouka C, Martinez-Laso J (1999). The origin of Cretan populations as determined by characterization of HLA alleles. Tissue Antigens.

[49] Kapustin S, Lyshchov A, Alexandrova J, Imyanitov E, Blinov M (1999). HLA class II molecular polymorphisms in healthy Slavic individuals from North-Western Russia. Tissue Antigens.

[50] Arnaiz-Villena A, Martinez-Laso J, Moscoso J, Livshits G, Zamora J, Gomez-Casado E, Silvera-Redondo C, Melvin K, Crawford M H (2003). HLA genes in the Chuvashian population from European Russia: admixture of Central European and Mediterranean populations. Hum. Biol.

[51] Gao X, Bhatia K, Trent R J, Serjeantson S W (1992). HLA-DR,DQ nucleotide sequence polymorphisms in five Melanesian populations. Tissue Antigens.

[52] Lester S, Cassidy S, Humphreys I, Bennett G, Hurley C K, Boettcher B, McCluskey J (1995). Evolution in HLA-DRB1 and major histocompatibility complex class II haplotypes of Australian aborigines. Definition of a new DRB1 allele and distribution of DRB1 gene frequencies. Hum. Immunol.

[53] Grahovac B, Sukernik R I, O'hUigin C, Zaleska-Rutczynska Z, Blagitko N, Raldugina O, Kosutic T, Satta Y, Figueroa F, Takahata N, Klein J (1998). Polymorphism of the HLA class II loci in Siberian populations. Hum. Genet.

[54] Gao X, Veale A, Serjeantson S W (1992). HLA class II diversity in Australian aborigines: unusual HLA-DRB1 alleles. Immunogenetics.

[55] Monsalve MV, Edin G, Devine D V (1998). Analysis of HLA class I and class II in Na-Dene and Amerindian populations from British Columbia, Canada. Hum. Immunol.

[56] Gao X, Zimmet P, Serjeantson S W (1992). HLA-DR,DQ sequence polymorphisms in Polynesians, Micronesians, and Javanese. Hum. Immunol.

[57] Cadavid L F, Watkins D I (1997). Heirs of the jaguar and the anaconda: HLA, conquest and disease in the indigenous populations of the Americas. Tissue Antigens.

[58] Middleton D, Menchaca L, Rood H, Komerofsky R (2003). New allele frequency database: http://www.allelefrequencies.net. Tissue Antigens.

[59] Dawkins R, Leelayuwat C, Gaudieri S, Tay G, Hui J, Cattley S, Martinez P, Kulski J (1999). Genomics of the major histocompatibility complex: haplotypes, duplication, retroviruses and disease. Immunol. Rev.

[60] Takahata N, Nei M (1990). Allelic genealogy under overdominant and frequency-dependent selection and polymorphism of major histocompatibility complex loci. Genetics.

[61] Bodmer WF, Bodmer JG (1978). Evolution and function of the HLA system. Br. Med. Bull.

[62] Martinez-Laso J, Moscoso J, Zamora J, Martín-Villa JM, Lowy E, Vargas-Alarcon G, Serrano-Vela JI, Gomez-Casado E, Arnaiz-Villena A (2004). Different evolutionary pathway of B*5701 and B*5801 (B17 group) alleles based in intron sequences. Immunogenetics.

[63] Cavalli-Sforza LL (1997). Genes, peoples, and languages. Proc. Natl. Acad. Sci. USA.

[64] Ruhlen M (1991). A guide of the world´s languages. I Classification.

[65] Dobbins F (1993). Disease transfer contact. Annu. Rev. Anthropol.

[66] Slade RW, McCallum HI (1992). Overdominance vs frequency-dependent selection at MHC loci. Genetics.

